# Parental Stress and Psychological Distress and Their Association With Spousal Support Among Parents of School-Age Children During the COVID-19 Pandemic in South India: A Cross-Sectional Observational Study

**DOI:** 10.7759/cureus.70372

**Published:** 2024-09-28

**Authors:** Sthuthi Shireen, Arya Jith, Priya E Thomas, Sharon P Methala, Kathleen A Mathew

**Affiliations:** 1 Psychiatry, Amrita Institute of Medical Sciences, Kochi, IND; 2 Psychiatry, Sree Uthradom Thirunal (SUT) Academy of Medical Sciences, Thiruvananthapuram, IND; 3 Psychiatry, Kasturba Medical College, Mangalore, IND; 4 Gastroenterology, Amrita Institute of Medical Sciences, Kochi, IND

**Keywords:** pandemic, parental stress, psychological distress, school-age children, spousal support

## Abstract

Context: The COVID-19 pandemic presented unprecedented challenges to families in the form of social isolation, economic difficulties and inaccessibility of educational and supportive services.

Aims: To assess the levels of parental stress and psychological distress among parents of school-age children during the COVID-19 pandemic and to analyse its correlation with spousal support.

Materials and methods: A cross-sectional, web-based study was conducted among the parents of school-age children in south India. One hundred thirty-two completed responses were obtained. Study tools included Parental Stress Scale, Spouse Support Scale and Kessler’s psychological distress scale-6 item version.

Results: The mean parental stress score was 42.39 ± 9.679. Sixty percent of the participants reported moderate to high levels of psychological distress. Parental stress and psychological distress scores were noted to have a significant negative correlation with spousal support scores (r=-.486, p=.000 and r=-.401, p=.000 respectively).

Conclusion: Our study findings show that the COVID-19 pandemic has taken a toll on the psychological well-being of parents of school-age children. Supportive interventions to address parenting difficulties need to be undertaken to prevent long-term adverse mental health outcomes. Lessons learnt from the pandemic are important for clinicians and policymakers to take measures to alleviate parental stress as well as to promote the psychological well-being of parents and children.

## Introduction

The novel coronavirus SARS-CoV2 outbreak created a state of public health emergency due to its far-reaching negative implications on the physical, socio-economic and psychosocial well-being of individuals. The prolonged home confinement imposed following the outbreak has contributed to stress within the community and family structure. Families are more likely to have experienced social isolation, economic difficulties and the inability to access educational and supportive services as a result. Specific recommendations like closing of schools and childcare agencies left parents in a vulnerable state having to face the demands of limiting social interaction and having to remain at home with their children [1].

A study conducted in Germany reported more than 50% of parents being stressed by social distancing and the closure of schools and childcare facilities [2], while a study conducted in India had 37% of parents reporting feeling more stressed as a parent after the lockdown [3]. Research has also shown an association between parenting stress and the perceived impact of COVID-19 among parents [4]. Previous studies indicate an increased risk of child abuse, childhood obesity [5], externalizing behavioural problems [6], dysfunctional parent-child interactions including lower parenting sensitivity [7], and higher prevalence of dysfunctional attachment styles, associated with greater parenting stress.

Co-parent relationship distress has also been considered as a risk factor for dysfunctional parent-child interactions and reduced proactive parenting. Research states that the impact of parental relationship distress on parent-child functioning during this time would be immense given that young children are likely to be more exposed to parental interactions while receiving less/no out-of-home care [8]. In another study, 9% of parents reported having developed interpersonal issues with their spouse while 35% reported an improved relationship with their partner, which was found to be associated with lower parental stress [3].

Parental adjustments during emergencies are important predictors of mental health outcomes of children [9]. Hence, it is absolutely essential to evaluate the mental health status of all parents/caregivers in the wake of the pandemic in order to prevent any detrimental outcomes. Findings from a study in Italy, one of the worst hit countries by COVID-19, highlighted an urgent need to provide parents with adequate support to take care of their own psychological well-being and to help their children cope with the direct and indirect effects of the pandemic [10].

In our study, we plan to focus on exploring parenting stress and psychological distress experienced by parents of school-going children in the midst of the lifestyle changes brought about by the public health measures put in place in India to mitigate the transmission of COVID-19. We also aim to study the role spousal support had to play on the psychological status of parents, an important resource of support for parents during the pandemic, about which to the best of our knowledge, limited research is available. We hope to gain information from our study that would assist in planning necessary interventions for families which need to be delivered in a timely manner in our community.

## Materials and methods

Setting and design

This was a cross-sectional, descriptive web-based study conducted among the parents of school-going children in south India. Ethical clearance was obtained from the Ethics Committee of Amrita School of Medicine (approval ECASM-AIMS-2021-309) prior to the commencement of the study. The study duration extended over two months (January and February 2022).

Sample size calculation

Based on the results of mean and standard deviation (SD) of parental stress score observed in an earlier publication [3], and with 15% allowable error and 95% confidence, the minimum sample size came to 10.

Inclusion and exclusion criteria

Both fathers and mothers from south India with children between five and 16 years of age were included in the study. In the case of multiple children, the parent was asked to report on one child only. A web-based survey for data collection including socio-demographic details and the study tools was created and the link was circulated among the contacts of the investigators meeting the inclusion criteria as well as on social media groups. Informed consent was obtained from all participants.

Study tools

Parental Stress Scale (PSS)

This is an 18-item self-report scale representing positive and negative themes of parenthood. It is a five-point scale (strongly disagree, disagree, undecided, agree, strongly agree) where respondents choose an option based on their typical relationship with their children. Eight items are reverse scored so that possible scores on the scale range between 18 and 90. It was developed by Berry and Jones in 1995 as an alternative to the 101-item Parenting Stress Index and can be used to assess changes in parental stress levels. Lower scores are indicative of lower levels of parental stress while higher scores point to high levels of stress. PSS has shown satisfactory levels of Cronbach’s alpha in previous studies (α = .75) [11].

Spouse Support Scale

This is a 10-item four-point (1=strongly agree, 2=agree, 3=disagree, 4=strongly disagree) scale that reflects respondents’ satisfaction with the degree of assistance spouses provide for child-rearing. It is a subscale of the larger Cleminshaw-Guidubaldi Parent Satisfaction Scale developed in 1985 by John Guidubaldi and Helen K Cleminshaw. Items 1, 2, 3, 4, 5, 7 and 8 are reverse scored (4=strongly agree to 1=strongly disagree). The item scores are then summed up and 10 is subtracted from the resulting score. The final score thus obtained ranges from 0 to 30. Higher scores indicate greater perceptions of spousal support. This scale has been found to be a reliable instrument with an internal consistency of 0.93 [12].

Kessler’s Psychological Distress Scale-6 Item Version (K6)

This is a six-item five-point scale with scores for individual responses to each item ranging from 0 for “none of the time” to 4 for “all of the time”. This scale is employed to quantify non-specific psychological distress. It strongly discriminates between community cases and non-cases of the Diagnostic and Statistical Manual of Mental Disorders, Fourth Edition (DSM-IV) disorders. The total scores range from 0 to 24 with higher scores reflecting higher levels of psychological distress and serious mental illness; a score of 0-7 indicates low distress, 8-12 moderate distress, and 13 to 24 is considered as high risk of psychological distress. K6 scale has been found to have adequate reliability in previous studies with Cronbach’s alpha ranging from 0.89 to 0.92 [13].

Statistical analysis

Data obtained was analysed using SPSS version 20.0 software (IBM Corp., Armonk, NY, USA). Continuous variables were represented as mean ± SD. To test the statistical significance of the difference in mean values of PSS and K6 scores with socio-demographic variables, independent samples t-test (for normal data) and Mann-Whitney U test (for non-normal data) were applied. Pearson correlation was employed to test the statistical significance of the correlation of spousal support with parental stress and psychological distress. The level of significance was P<0.05.

## Results

Our study included 132 participants, with the majority being females (84.1%). The mean age of the participants was 38.48 ± 5.456 years. 74.2% of them had postgraduate or higher levels of education. 98.5% of them were married and most of them lived in nuclear families (73.5%). In the majority of the cases, both parents were employed and had to work outside the home (43.2%). The study subjects belonged to the states of Kerala, Karnataka and Tamil Nadu. 11.4% of the subjects had medical comorbidities and 7.6% reported psychiatric comorbidities. 28.8% of the participants reported having experienced financial constraints during the pandemic. 9.8% of the children were reported to have neurodevelopmental issues (ADHD (3.8%), autism spectrum disorder (0.8%), specific learning disability (1.5%), anxiety/depression (2.3%), and other behavioural problems (1.5%)) (Table 1).

**Table 1:** Socio-demographic and clinical profile of the participants (n=132)

Variables	Categories	n (%)
Gender	Male	21(15.9)
	Female	111 (84.1)
Marital status	Married	130 (98.5)
	Separated/divorced	2 (1.5)
Highest educational qualification	Graduate	34 (25.8)
	Post-graduate or higher	98 (74.2)
State of residence in India	Kerala	117 (88.6)
	Karnataka	9 (6.8)
	Tamil Nadu	6 (4.5)
Family type	Nuclear	97 (73.5)
	Joint	35 (26.5)
Employment status	Both parents work outside home	57 (43.2)
	Both parents work from home	23 (17.4)
	Only one parent employed	48 (36.4)
	Both parents unemployed	4 (3)
Medical co-morbidities	Yes	15 (11.4)
	No	117 (88.6)
Psychiatric co-morbidities	Yes	10 (7.6)
	No	122 (92.4)
Financial difficulty during the pandemic	Yes	38 (28.8)
	No	94 (71.2)
Number of children	One child	45 (34.1)
	Two or more children	87 (65.9)
Neurodevelopmental/psychiatric disorders in the child	Yes	13 (9.8)
	No	119 (90.2)

A high percentage (71.2%) of parents reported that closure of schools and introduction of online classes was stressful for them. 55.3% of the parents reported that they considered reopening of schools as less stressful compared to online classes.

The mean parental stress score was noted to be 42.39 ± 9.679. The mean score on Spouse Support Scale was 27.52 ± 6.836. A high level of psychological distress was reported by 24.2% of the participants while 36.4% reported moderate levels of distress (Table 2).

**Table 2:** Level of psychological distress among the participants

Level of psychological distress	n (%)
Low	52 (39.4)
Moderate	48 (36.4)
High	32 (24.2)

Individuals who were below 40 years of age were noted to have significantly higher levels of parental stress as compared to those above 40 years of age (p=0.006). Women were noted to have higher parental stress levels as compared to men, though the difference did not reach statistical significance (p=.068) (Table 3). No significant association was found between psychological distress and socio-demographic variables (Table 4).

**Table 3:** Association of socio-demographic and clinical variables with levels of parental stress (n=132) Independent samples t-test was used to find the p values *P value significant at <0.05

Respondent variables		Parental stress		
Variable	Category	Mean (SD)	t	p
Age	<40 years (n=85)	44.08 (9.330)	2.776	.006*
	≥ 40 years (n=47)	39.32 (9.639)		
Gender	Male (n=21)	38.86 (8.027)	1.839	.068
	Female (n=111)	43.05 (9.851)		
Highest educational qualification	Graduate (n=34)	44.97 (9.100)	1.823	.071
	Postgraduate or higher (n=98)	41.49 (9.757)		
Family type	Nuclear (n=97)	42.16 (9.055)	.436	.663
	Joint (n=35)	43.00 (11.355)		
Medical comorbidity	Yes (n=15)	45.93 (10.872)	1.515	.132
	No (n=117)	41.93 (9.471)		
Psychiatric comorbidity	Yes (n=10)	45.90 (4.886)	1.196	.234
	No (n=122)	42.10 (9.927)		
Financial difficulty during COVID-19	Yes (n=38)	42.21 (8.814)	.132	.895
	No (n=94)	42.46 (10.052)		
Number of children	One child (n=45)	42.31 (11.937)	.017	.987
	Two or more children (n=87)	42.34 (8.339)		

**Table 4:** Association of socio-demographic and clinical variables with levels of psychological distress (n=132) Mann Whitney U test was used to find the p values *P value significant at <0.05

Respondent variables		Psychological distress		
Variable	Category	Mean rank	U	p
Age	<40 years (n=85)	71.04	1611.500	.066
	≥ 40 years (n=47)	58.29		
Gender	Male (n=21)	53.79	898.500	.096
	Female (n=111)	68.91		
Highest educational qualification	Graduate (n=34)	70.94	1515.000	.431
	Postgraduate or higher (n=98)	64.96		
Family type	Nuclear (n=97)	67.52	1598.500	.609
	Joint (n=35)	63.67		
Medical comorbidity	Yes (n=15)	65.87	868.000	.946
	No (n=117)	66.58		
Psychiatric comorbidity	Yes (n=10)	88.20	393.000	.061
	No (n=122)	64.72		
Financial difficulty during COVID-19	Yes (n=38)	70.82	1622.000	.408
	No (n=94)	64.76		
Number of children	One child (n=45)	65.24	1654.000	.520
	Two or more children (n=87)	60.94		

Parental stress score was found to have a significant negative correlation with spousal support scores (r= -.486, p=.000). This indicates that the lesser the spousal support, the greater the levels of parental stress. Similarly, psychological distress score was negatively correlative with spousal support and this association was statistically significant (r= -.401, p=.000) which shows that greater levels of psychological distress were seen in those individuals who had poor spousal support (Table 5).

**Table 5:** Correlation of parental stress scores and psychological distress scores with spousal support scores *Correlation is significant at the 0.01 level (2-tailed)

Variables	Spousal support score	
	r	p
Parental stress	-.486	.000*
Psychological distress	-.401	.000*

## Discussion

The COVID-19 pandemic posed an unprecedented threat to the psychological well-being of parents across the globe. Our main aim in this study was to investigate the impact of the pandemic on parental stress and psychological distress levels. We also aimed to examine whether spousal support had a role in mitigating the deleterious effects on parental mental health.

Our study findings showed that a large proportion of parents (60%) experienced moderate to high levels of psychological distress during the pandemic. A majority (71.2%) of the parents reported that closure of schools and introduction of online mode of classes was stressful for them. These findings are in line with existing research which shows that the threat posed by the pandemic has had significant implications on the mental well-being of parents. A study conducted among 196 Indian parents reported that 67% of the parents had moderate to high levels of perceived stress [3]. Many parents had to deal with working from home while caring for their children, creating structures and routines to guide children’s learning at home, amidst other burdens like reduced wages and other financial difficulties [14]. Parents were often forced to multitask in the roles of caregiver, teacher, playmate and entertainer to their children in addition to fulfilling professional responsibilities, which may have added to their emotional burden [15].

Research has consistently demonstrated a positive relationship between social support and psychological well-being of an individual [16]. One possibility is that the perception of being supported by close ones can encourage the use of adaptive coping strategies by an individual in the wake of stress, thereby promoting mental well-being. The richness or scarcity of naturally available resources and social support play a key role in deciding parenting behaviour. In situations such as the COVID-19 crisis, when most sources of social support were inaccessible following the social distancing and lock-down norms, spouses had an important role as the primary source of support. However only a few studies have looked into this important resource of support and its role in buffering the negative effects of stress. Spousal support refers to a form of family support comprising enriching experiences derived as a result of a spouse stepping in to take up responsibilities such as household chores or listening to one’s stressful experiences such as those related to work. A notable finding of our study is the negative correlation between spousal support and levels of parental stress and psychological distress. This is in agreement with the findings of a study involving 511 Turkish couples which showed that perceived stress score was negatively correlated with spousal support and marital satisfaction [17].

The Systemic Transaction Model (STM) by Bodenmann (1995) is amongst the first theories that conceived stress and coping responses as social processes that are ingrained in close relationships, with a special focus on interdependence between partners [18]. Stressors always affect both partners involved in an intimate relationship either by primarily affecting one partner, where his/her stress and coping spills over to the other partner, or by simultaneously affecting both partners (shared stressors such as the pandemic) [19]. A central concept of this theory is ‘dyadic coping’ which refers to a couple’s capacity to deal with external stressors with the aim of maintaining the well-being of both partners by reducing the levels of stress experienced by each individual and mutually promoting adequate functioning [20]. Three positive forms of dyadic coping have been described, namely, supportive dyadic coping (helping one’s partner find solutions and lending emotional support), delegated dyadic coping (relieving the partner of responsibilities) and common dyadic coping (both partners work together through the stressor). Negative dyadic coping refers to reacting to one’s partner’s stress with hostility, indifference or ambivalence. Putney et al. observed that supportive dyadic coping was associated with lesser parenting stress in a study conducted among 184 couples who had a child with autism spectrum disorder [21]. This model can be employed to elucidate the implications of the link between spousal support and parental psychological impact in our study.

Participants aged less than 40 years were observed to have significantly higher levels of parental stress as compared to those aged 40 or above. This group of parents being younger, may be less experienced as parents and may have more difficulty coping in the role of primary caregivers in the midst of a crisis situation [22]. These parents are also more likely to have younger school-age children who may require more attention and assistance than older children, which may also contribute to heightened levels of parenting stress. Early results from the UK Co-SPACE study indicate that parents who had young children (10 years or less) were more likely to report being unable to meet the demands of their work and the needs of their child as compared to parents with children aged 11 years or above (56.2% versus 45.8%) [23].

Our findings have crucial implications for both policy and practice. Parents of school-age children have faced unparalleled levels of psychological distress and heightened parental stress during the pandemic. Parents need to be educated and sensitised about parental stress and its impact on the child’s developmental and emotional outcomes. Skill-based interventions that promote positive coping strategies and problem-solving skills should be implemented, targeting high-risk families at the community level and preventive public health measures focussing on parenting-specific challenges need to be instituted. Health organisations like the World Health Organisation and Centers for Disease Control and Prevention have made efforts to provide online resources to address parenting difficulties and evidence-based self-help strategies to effectively manage stress during COVID-19. Further research is required to assess whether the general public is able to access and effectively make use of these resources. Policymakers at the local and national level should plan collaboratively with schools and mental health practitioners to plan coordinated strategies to mitigate the detrimental impact of COVID-19 on families. This would also help improve our preparedness in the event of community disasters.

Limitations

This study had certain limitations. Since we conducted a web-based survey, self-selection bias is likely to have occurred. Our sample consisted of parents who had a graduate level of education and above, and were individuals who had access to internet. Hence the findings of this study may not be generalizable to the population. Many parents had two or more children in the school-going age group due to which we were unable to analyse the role of age, gender and other child-related variables on parenting stress.

## Conclusions

Our findings lend scientific credence to and validate the challenges experienced by parents during the COVID-19 pandemic. It demonstrates the toll that the pandemic has taken on the psychological well-being of parents as they attempted to juggle home-schooling and parenting with multiple other responsibilities. In the event of community disasters, when external social support systems are inaccessible, spousal support assumes paramount importance. It is vital to institute appropriate supportive measures and policies to alleviate the negative psychological effects on parents and thereby prevent adverse child mental and physical health outcomes.
